# Custom-Built Operant Conditioning Setup for Calcium Imaging and Cognitive Testing in Freely Moving Mice

**DOI:** 10.1523/ENEURO.0430-21.2022

**Published:** 2022-02-09

**Authors:** Philip Vassilev, Esmeralda Fonseca, Giovanni Hernandez, Andrea Haree Pantoja-Urban, Michel Giroux, Dominique Nouel, Elise Van Leer, Cecilia Flores

**Affiliations:** 1 Department of Psychiatry and Department of Neurology and Neurosurgery, McGill University, Montréal, QC, H3A 1A1, Canada; 2 Douglas Mental Health University Institute, Montreal, QC, H4H 1R3, Canada; 3 Princeton Neuroscience Institute, Princeton University, Princeton, NJ 08540; 4 Integrated Program in Neuroscience, McGill University, Montréal, QC, H3A 1A1, Canada

**Keywords:** animal models, calcium imaging, cognition, open-source, operant conditioning

## Abstract

Operant chambers are widely used in animal research to study cognition, motivation, and learning processes. Paired with the rapidly developing technologies for brain imaging and manipulations of brain activity, operant conditioning chambers are a powerful tool for neuroscience research. The behavioral testing and imaging setups that are commercially available are often quite costly. Here, we present a custom-built operant chamber that can be constructed in a few days by an unexperienced user with relatively inexpensive, widely available materials. The advantages of our operant setup compared with other open-source and closed-source solutions are its relatively low cost, its support of complex behavioral tasks, its user-friendly setup, and its validated functionality with video imaging of behavior and calcium imaging using the UCLA Miniscope. Using this setup, we replicate our previously published findings showing that mice exposed to social defeat stress in adolescence have inhibitory control impairments in the Go/No-Go task when they reach adulthood. We also present calcium imaging data of medial prefrontal cortex (mPFC) neuronal activity acquired during Go/No-Go testing in freely moving mice and show that neuronal population activity increases from day 1 to day 14 of the task. We propose that our operant chamber is a cheaper alternative to its commercially available counterparts and offers a better balance between versatility and user-friendly setup than other open-source alternatives.

## Visual Abstract

**Figure:**
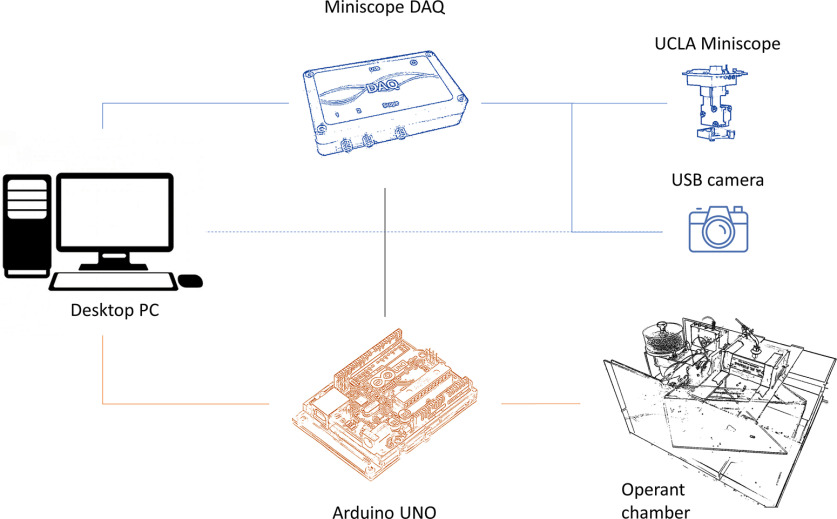

## Significance Statement

Operant conditioning chambers are widely used in neuroscience research, but commercially available operant setups are often costly. Here, we describe the construction of an open-source, low-cost operant conditioning setup which can be constructed in a few days using widely available materials. Using this setup, we replicate our previous findings showing that exposure to social defeat stress in adolescence impairs inhibitory control in adulthood. We also pair our setup with *in vivo* calcium imaging, and we show that we can record calcium activity from individual neurons in the medial prefrontal cortex (mPFC) of freely moving mice while they carry out a cognitive task. Our custom-built operant conditioning setup is a useful tool to study the neurobiology of both adaptive and pathologic behavior.

## Introduction

Operant conditioning chambers (“Skinner boxes”) are widely used in animal research to study learning and other cognitive processes in rodents, some birds, and nonhuman primates (Panlilio and Goldberg, 2007; Paglieri et al., 2014). An operant chamber allows for precisely controlled presentation of stimuli (auditory, visual, etc.) and for the measurement of responses to these stimuli (e.g., lever presses or nose pokes). By controlling the presentation of stimuli and their relationship to the responses of test subjects, experimenters can design behavioral tasks that assess motivation, learning (operant and classical conditioning) or other cognitive processes (e.g., attention, inhibitory control, etc.). Optogenetic tools and fluorescent biosensors for calcium imaging have made it possible to record and control brain activity during such behavioral tasks to identify neural circuits involved in overt behavior (Bernstein and Boyden, 2011; Knopfel, 2012). This combined approach is the basis of many preclinical studies investigating behavioral traits of model organisms that resemble symptoms observed in psychiatric disease in humans and has provided important insight into the neurobiological basis, potential treatment, and prevention strategies for psychiatric disorders (Tye and Deisseroth, 2012; Siciliano and Tye, 2019).

As the technologies employed in behavioral and brain-imaging studies have improved, their associated costs have increased significantly, the commercially available conditioning and imaging setups have become much more sophisticated but also much more expensive. Researchers often need to invest a lot in equipment which comes with additional expenses, proprietary software, high maintenance costs, costly updates, and additional features. Therefore, the interest in open-source platforms and tools is gaining increasing popularity (Freeman, 2015; White et al., 2019). Many scientific laboratories now produce their own behavioral platforms, brain imaging systems and data analytic tools to address specific research questions (Pineno, 2014; Freeman, 2015; Devarakonda et al., 2016; Kokras et al., 2017; O’Leary et al., 2018; Ribeiro et al., 2018; Erskine et al., 2019; Gurley, 2019; Buscher et al., 2020; Lee et al., 2020; Mazziotti et al., 2020; Tran et al., 2020; Vassilev et al., 2020; Matikainen-Ankney et al., 2021).

Here, we present the procedure for building an open-source Arduino-based operant chamber paired with an *in vivo* calcium imaging platform, the UCLA Miniscope (Cai et al., 2016; Aharoni and Hoogland, 2019; Aharoni et al., 2019), to assess medial prefrontal cortex (mPFC) neuronal activity of freely moving mice while they perform the Go/No-Go task. Our operant setup is relatively low-cost, which makes it preferable to commercially available alternatives. Compared with open-source alternatives, it has a wider range of programmable stimuli (two auditory and two visual), it offers the code and setup of a complex behavioral task with two measures of impulsivity, as well as attention, and it has a relatively user-friendly setup with fewer electronic components and less programming. Finally, it has validated functionality with video imaging of behavior and calcium imaging.

We demonstrate the efficacy of our setup by reproducing our previous findings that exposure to accelerated social defeat stress (AcSD) in adolescence leads to impaired Go/No-Go performance in adulthood (Vassilev et al., 2021). The Go/No-Go task measures the ability to withhold a previously learned response which is no longer appropriate (i.e., inhibitory control or motor impulsivity), and neurons of the mPFC have been shown to encode information in this task (Pinto and Dan, 2015; Kamigaki and Dan, 2017; Li et al., 2020). Using our setup, we provide calcium imaging data showing changes in population activity of mPFC neurons while male mice are performing early versus late stages of the task. The Go/No-Go task has an equivalent in both rodents and humans, and impairments in the task, as well as abnormal mPFC activity, are characteristic traits of several psychiatric disorders with adolescent onset (Weisbrod et al., 2000; Kaiser et al., 2003; Reynolds et al., 2018). We propose that our setup is an affordable and versatile tool for preclinical and translational research into the neural substrates of both adaptive and pathologic behavior.

## Materials and Methods

### Animals

Experimental procedures were performed in accordance with the guidelines of the Canadian Council of Animal Care and approved by the McGill University and Douglas Hospital Animal Care Committee. All mice were housed in a temperature-controlled and humidity-controlled (21–22°C; 60%) colony room of the Neurophenotyping center of the Douglas Mental Health University Institute, on a 12/12 h light/dark cycle (light on at 8 A.M.). The mice had *ad libitum* access to food and water throughout the experiments (except during food restriction for Go/No-Go experiments). Mice were assigned randomly to each experimental condition.

Male C57BL/6J wild-type mice (*n *=* *27) supplied by The Jackson Laboratory, arrived at the housing facilities on postnatal day (PND)24. These mice were housed in groups of three to four animals per cage before exposure to AcSD and single-housed after AcSD. Male CD-1 retired breeder mice (more than three months of age) obtained from Charles River Canada were used as aggressors in the AcSD paradigm.

The present study is aimed primarily at validating the custom-built behavioral and imaging setup, so only male mice were used as subjects. Although sex differences in the effects of adolescent social stress are very likely and of considerable interest, they are outside the scope of the present article.

### AcSD paradigm

The AcSD procedure was conducted as described previously (Vassilev et al., 2021). The AcSD apparatus consisted of a transparent rat cage with two mouse housing compartments separated by a transparent and perforated central divider that allowed sensory but not physical contact between mice. Before the AcSD sessions, aggressor CD-1 mice were screened for aggression toward adolescent C57BL/6J mice. Then, they were housed on one side of the divider, where defeat sessions took place. Adolescent C57BL/6J mice were exposed to the aggressive CD-1 mice for two sessions per day for a total of 4 d (between PND25 and PND28; “defeat” group). Following defeat sessions, experimental mice were housed on the empty side of the cage divider until the next session. To ensure aggressive behavior toward adolescent mice, the CD-1 mouse aggressors were primed for aggression by a brief (30 s) exposure to an adult (PND65) C57BL/6 mouse before each AcSD session. Twenty-four hours after the last session of AcSD, C57BL/6J adolescent mice were assessed in the social interaction test (SIT) to measure their approach and/or avoidance behavior toward an unfamiliar CD-1 mouse. We calculated an interaction ratio (IR) based on the ratio between the time spent in the interaction zone (IZ) in the presence and the absence of an unfamiliar CD-1 mouse. Mice with ratios >/= 1 were classified as “resilient” and < 1 – as “susceptible.”

### Calcium imaging

#### GRIN implantation and viral injection surgery

GRIN lens implantation and virus injection were done in the same surgical session. Mice were anaesthetized with isoflurane (5% induction and 1–2% maintenance) and placed on a stereotaxic frame. Eye ointment was applied to keep the eyes from drying. Intraperitoneal injections of saline and carprofen were administered to maintain hydration and to reduce inflammation. The skull was exposed by making a circular excision of the skin and connective tissue from between the eyes up to between the ears of the mouse. Two screws were secured to the back of the skull, one in each parietal bone, with the help of a microdrill and a screwdriver. A third whole (1.1 mm in diameter) was drilled in the skull just above the mPFC [centered at anterior-posterior (AP) +1.7 mm and medial-lateral (ML) +0.5 mm from bregma]. A 5-μl microsyringe was used to inject 0.3 μl of AAV9-Syn-GCaMP6f-WPRE virus (AddGene), over 6 min, in the mPFC [AP +1.7 mm, ML +0.5 mm, dorsal-ventral (DV) −1.8 mm]. After 10 min, the needle was extracted from the brain and a 1 × 4 mm GRIN lens (GoPhoton) held by a vacuum holder was inserted through the same hole in the skull that was used for the viral injection. The bottom of the GRIN lens was positioned at the same coordinates as the viral injection. We did not aspirate brain tissue because, in our experience, the hole made by the viral injection in the brain is sufficient to make way for the GRIN implantation. The GRIN lens was secured to the skull using glue and dental cement. The top of the lens was protected by placing a small protective cap made from the bottom of a PCR tube, secured by silicone (KwikSil, World Precision Instruments).

Three weeks later, mice were anaesthetized again, and an aluminum baseplate was secured to the head using dental cement to allow for the attachment of the miniature epifluorescent endoscope (UCLA Miniscope v3).

#### Recording and analysis of calcium transients

For the sake of consistency across sessions, the mice carried the miniscope during every session of the Go/No-Go task, from day 1 of training until the last day of testing. However, recording was done only during the testing phase (last 14 d). The recording of calcium transients was done using the open-source software of the UCLA Miniscope (Miniscope DAQ software 1.0, GitHub version August 30, 2020), at 20 frames per second. We recorded for the length of the whole 30 min Go/No-Go sessions. Cell detection and calcium trace extraction was achieved using the Suite2p package (Pachitariu et al., 2017). Signal drift across the session because of bleaching was corrected using a sliding window method (Pinto and Dan, 2015). To visualize neuronal activity in response to the presentation of “Go” and “No-Go” cues, we calculated the z-scored change in fluorescence in all detected neurons (regardless of significant or non-significant changes in activity) as well as cue-modulated neurons only and plotted the median fluorescence across trials. The custom MATLAB scripts are available on reasonable request.

### Go/No-Go task

To test cognitive function, we used the Go/No-Go task as previously described (Reynolds et al., 2018; Cuesta et al., 2020; Vassilev et al., 2021). The only difference from the previously described procedure was adding an initial training stage (for 3 d) where the active nose-poke hole was constantly illuminated until the mouse nose-poked. In this stage, every response led to reward delivery and a 10-s time out where the nose-poke light went off and responses were not rewarded. This stage was used for fine-tuning of the miniscope recording parameters. First, mice underwent AcSD and SIT in adolescence; ∼30 d later, in adulthood, mice were food restricted to 85% of free-feeding weight and started training for the behavioral task. Chocolate-flavored dustless precision pellets (BioServ) were used as a reinforcer. The task required mice to nose poke in response to an illuminated Go cue within a limited amount of time or inhibit their response to this cue when presented together with an auditory No-Go cue. Nose poke responses to the Go cue (“hits”) resulted in the delivery of a food pellet, while the same response to the No-Go cue was considered a “commission error” and resulted in reward omission. Responses during a 3- to 9-s window before cue presentation were considered premature and led to reward omission and trial restart. We also calculated a correct response rate representing the proportion of all available rewards that were acquired by mice on both Go and No-Go trials combined.

### Custom-built operant chambers and calcium imaging setup

**Figure 1.**
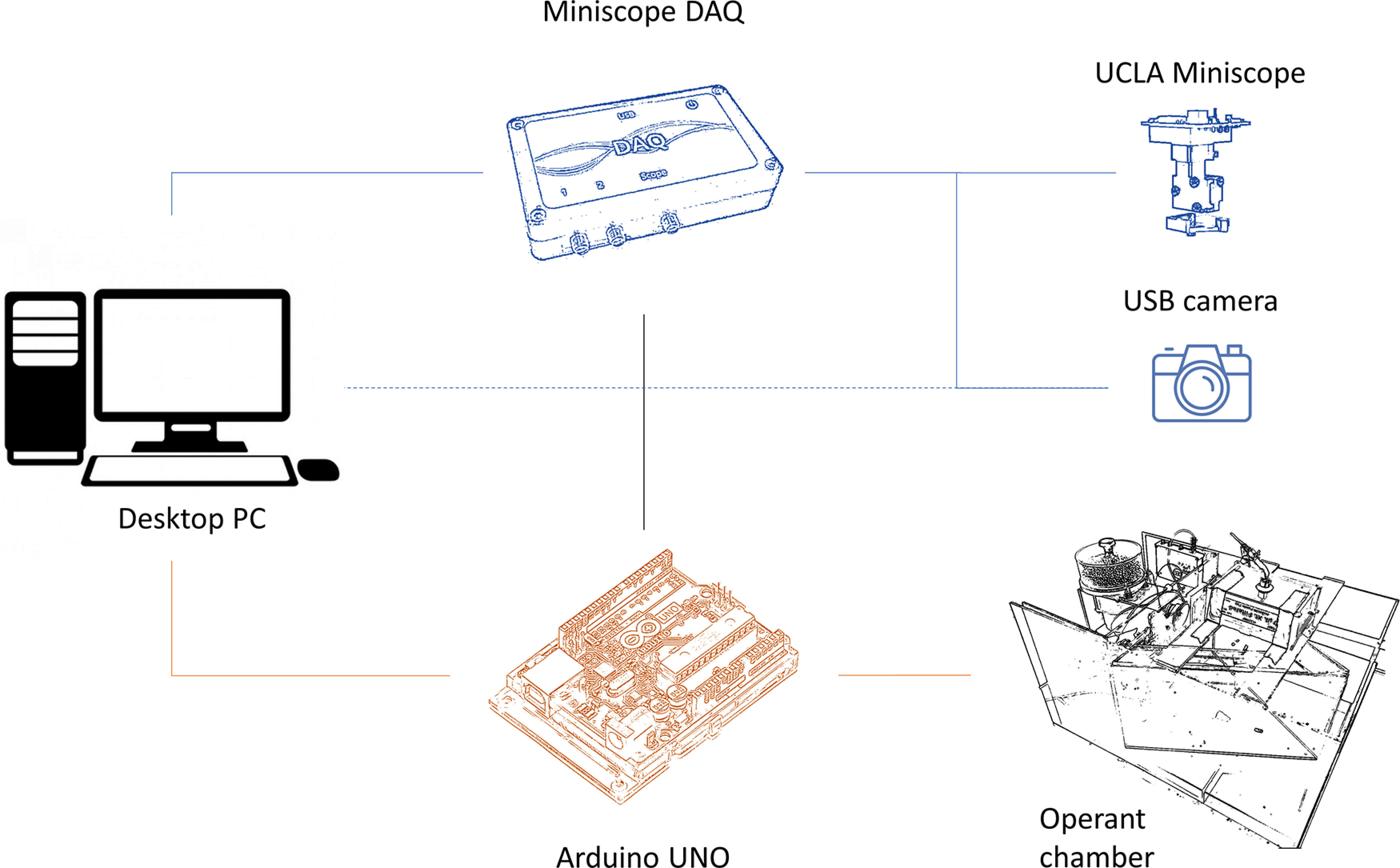
Overview of the setup. A desktop PC powers and controls an Arduino UNO microcontroller which in turn controls the nose poke sensors, food dispenser and audiovisual stimuli of the operant chamber. The Arduino and PC are also connected to the miniscope data acquisition box (DAQ). The DAQ, in turn, controls the USB camera for observation of the animal and the miniscope camera for recording of calcium activity. The USB camera is powered by the PC via a USB cable and the parameters of the recording can be manipulated both through the miniscope software and through other software installed for that purpose (e.g., ManyCam, Visicom Media Inc.).

All mice were trained and tested on the Go/No-Go task in custom operant chambers built on-site. The general layout of the setup is shown in Figure 1. A desktop PC connects to an Arduino UNO microcontroller which in turn controls the inputs and outputs of the operant chamber. The desktop PC also connects to the Data Acquisition Box (DAQ) of the UCLA Miniscope and to a USB camera for observation of mouse movement. Finally, the Arduino connects to the DAQ to initiate the recording of calcium activity through the miniscope.

#### Polycarbonate components of operant chambers

**Figure 2.**
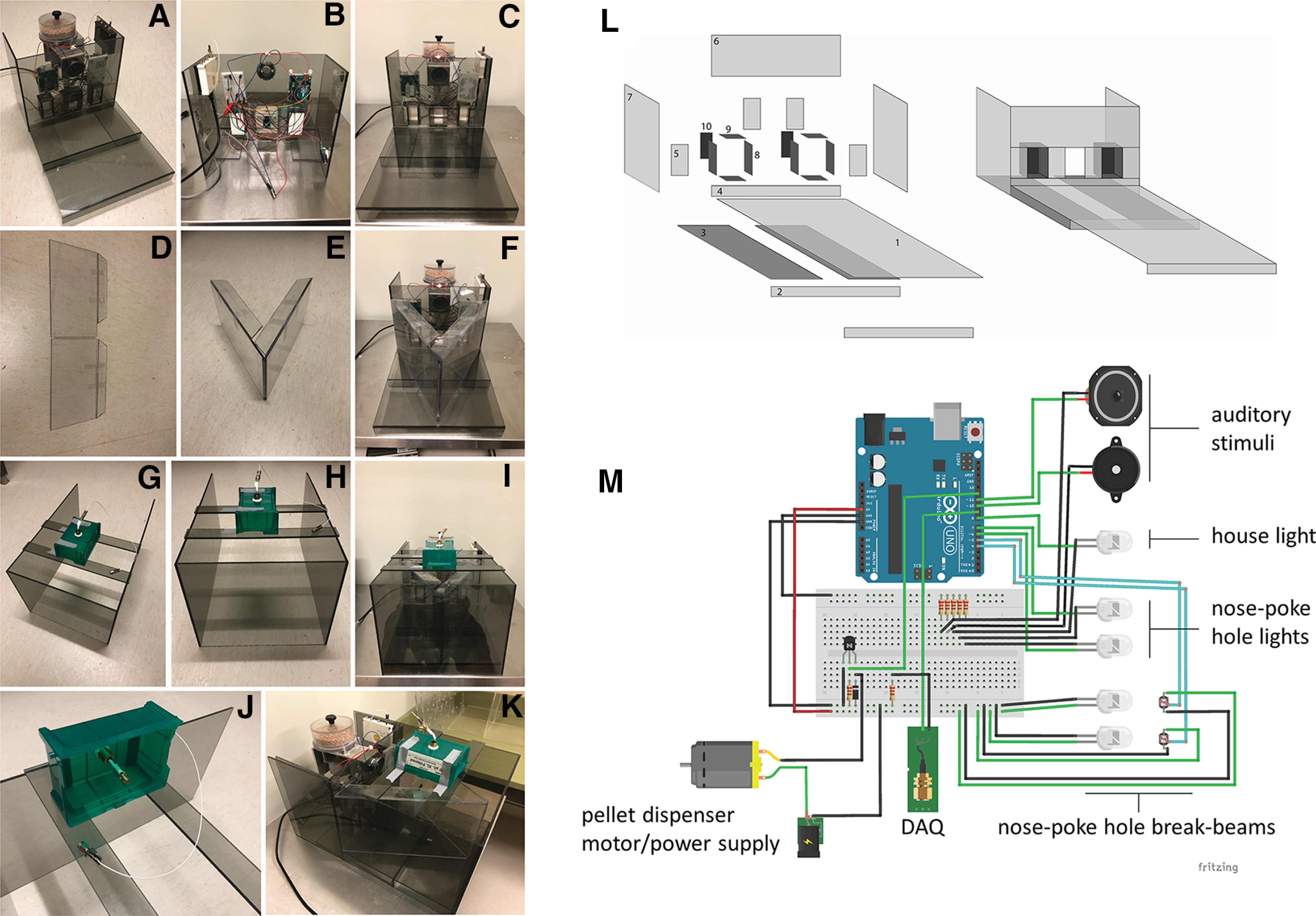
Operant chamber components. ***A–C***, The base of the operant chamber is composed of the floor and the front wall with two nose poke holes and an opening for the food receptacle (***A***). The back of this wall holds the electronic components (***B***) and the pellet dispenser (***C***). The individual polycarbonate pieces comprising the base of the chamber are shown in (***L***) and their dimensions are shown in Table 1; ***M*** represents a schematic of the electronic circuit controlling the sensors and stimuli of the chamber. ***D–F***, An additional set of removable walls limits the exploration of the mouse and focuses the behavior on the nose poke holes and pellet dispenser (optional). ***G–I***, A removable outer casing completes the box with outer walls and two strips above the box allow for the attachment of a pipette tip box with a commutator and the USB camera (data not shown; ***J***). The pipette box with the commutator is hinged using masking tape. ***K***, The operant chamber in its entirety. Extended Data Figure 2-1 shows the DAQ modification for connecting to the Arduino circuit, and Extended Data Figures 2-2, 2-3, 2-4, 2-5, 2-6, 2-7, 2-8, 2-9, 2-10, 2-11 contain the Arduino code for the Go/No-Go task.

The base and walls of the operant chambers were constructed using polycarbonate sheets (Fig. 2). We used plastics cement to attach individual polycarbonate pieces together to form the walls of the chamber. To allow removal and maintenance, we used white tac (UHU) or waterproof tape (e.g., Gorilla) to attach electronic components. The base of the chamber consists of a floor and a front wall with three identical openings, one for the food pellet receptacle and two for the nose-poke holes (Fig. 2*A–C*). Importantly, we designed the size of the nose-poke holes to accommodate for the size of the mouse head carrying a miniscope. The back of the wall carries the food dispenser, the Arduino, and all electronic components (Fig. 2*B*,*M*; described in detail below). We used the services of a local plastics store to cut the polycarbonate sheets which comprise the main body of the operant chambers (Fig. 2*L*). The dimensions of the individual polycarbonate pieces are provided in Table 1. A V-shaped polycarbonate barrier with overhangs forms the back walls of the testing chamber and prevents mice from jumping out during behavioral testing (Fig. 2*D–F*). Finally, the outside walls were constructed as a separate part of the chamber which can be removed to allow for cleaning (Fig. 2*G–I*). These outer walls have two thin polycarbonate pieces on top that carry a commutator (DragonFly Inc.) attached to a pipette box allowing free movement of the mouse carrying the miniscope (Fig. 2*J*).

**Table 1:** List of polycarbonate pieces, and their dimensions, necessary to build one operant chamber (as enumerated on Fig. 2*L***)**

Component #	Dimensions	Amount (per chamber)
1	11.375 × 11.375 inches	1
2	11.375 × 1.125 inches	2
3	11.875 × 3.25 inches	2
4	11.375 × 1.625 inches	1
5	2.375 × 2 inches	4
6	11.375 × 5.75 inches	2
7	9.75 × 5.875 inches	2
9	1.625 × 1.125 inches	6
8 and 10	2.375 × 1.125 inches	6
Outer enclosure	18.125 × 9.75 inches	2
Outer enclosure	11.5 × 9.75 inches	1
Outer enclosure	12 × 1.25 inches	2

10.1523/ENEURO.0430-21.2022.f2-1 Extended Data Figure 2-1 Miniscope DAQ modification for external triggering of recording. ***A***, ***B***, A coaxial cable with an SMA connector is soldered to the input port (red square) right next to the miniscope input. ***C***, A second coaxial cable is connected via SMA and a coaxial-to-SMA PCB. The free end is adapted to fit a breadboard by soldering two pins to the PCB. ***D***, A top view of the operant chamber showing the miniscope DAQ (red square) connected to the miniscope via the coaxial cables (white) and the commutator. The placement of the USB camera is also visible. Download Figure 2-1, TIF file.

10.1523/ENEURO.0430-21.2022.f2-2 Extended Data Figure 2-2 Arduino script for Go/No-Go training stage 0, left nose-poke hole active. Download Figure 2-2, TXT file.

**Figure 3.**
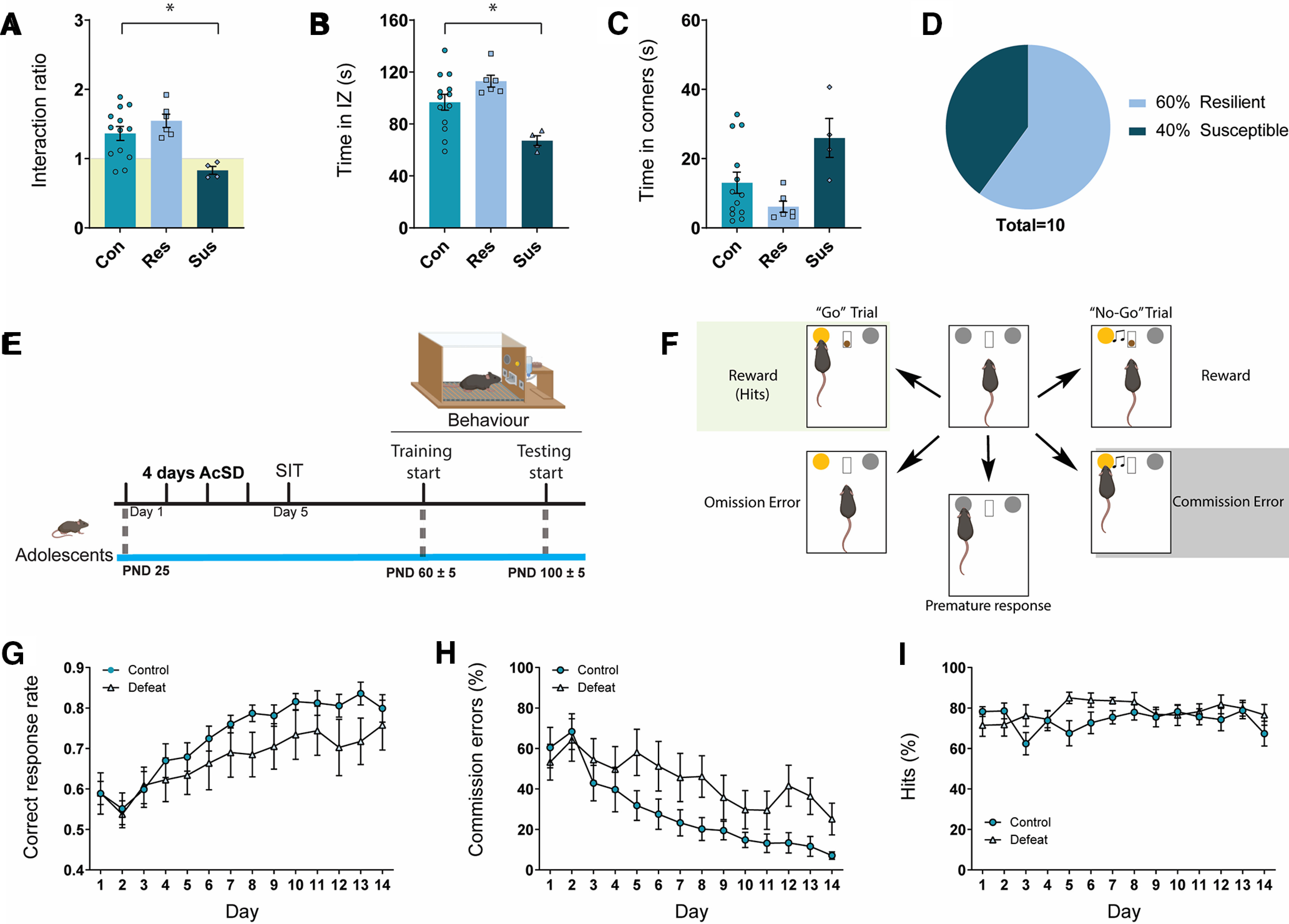
Validation of the operant setup – assessing the effect of adolescent social stress on cognition in adulthood. Adolescent mice were exposed to 4 days of accelerated social defeat stress (AcSD) and separated into resilient and susceptible groups based on a social interaction test (SIT). ***A***, Susceptible mice had interaction ratios (IR) < 1, while resilient mice had IR >/= 1. * significantly different from control, p = 0.01. ***B***, Susceptible mice spent less time in the interaction zone (IZ) of the SIT arena - avoiding an unfamiliar CD-1 mouse - and spent more time in the corner zones of the SIT arena (***C***) * significantly different from control, *p* = 0.02. ***D***, The majority (60%) of mice exposed to AcSD in adolescence were resilient. ***E***, One month after AcSD and SIT, in adulthood, mice were first trained and then tested on the Go/No-Go task. ***F***, Schematic representation of the Go/No-Go task. Mice receive rewards by nose-poking on “Go” trials (“hits”) and withholding a nose poke during No-Go trials. Commission errors represent incorrect responses on No-Go trials. Responses before the Go (light) or No-Go (light + tone) cues on any trial are considered “premature” responses and not rewarded. ***G***, The correct response rate represents the proportion of available rewards that were acquired by control and defeated mice across both Go and No-Go trials. ***H***, Defeated mice made more commission errors during No-Go trials. ***I***, There were no differences between control and defeated mice in the number of correct responses on Go trials.

10.1523/ENEURO.0430-21.2022.f2-3 Extended Data Figure 2-3 Arduino script for Go/No-Go training stage 0, right nose-poke hole active. Download Figure 2-3, TXT file.

**Figure 4.**
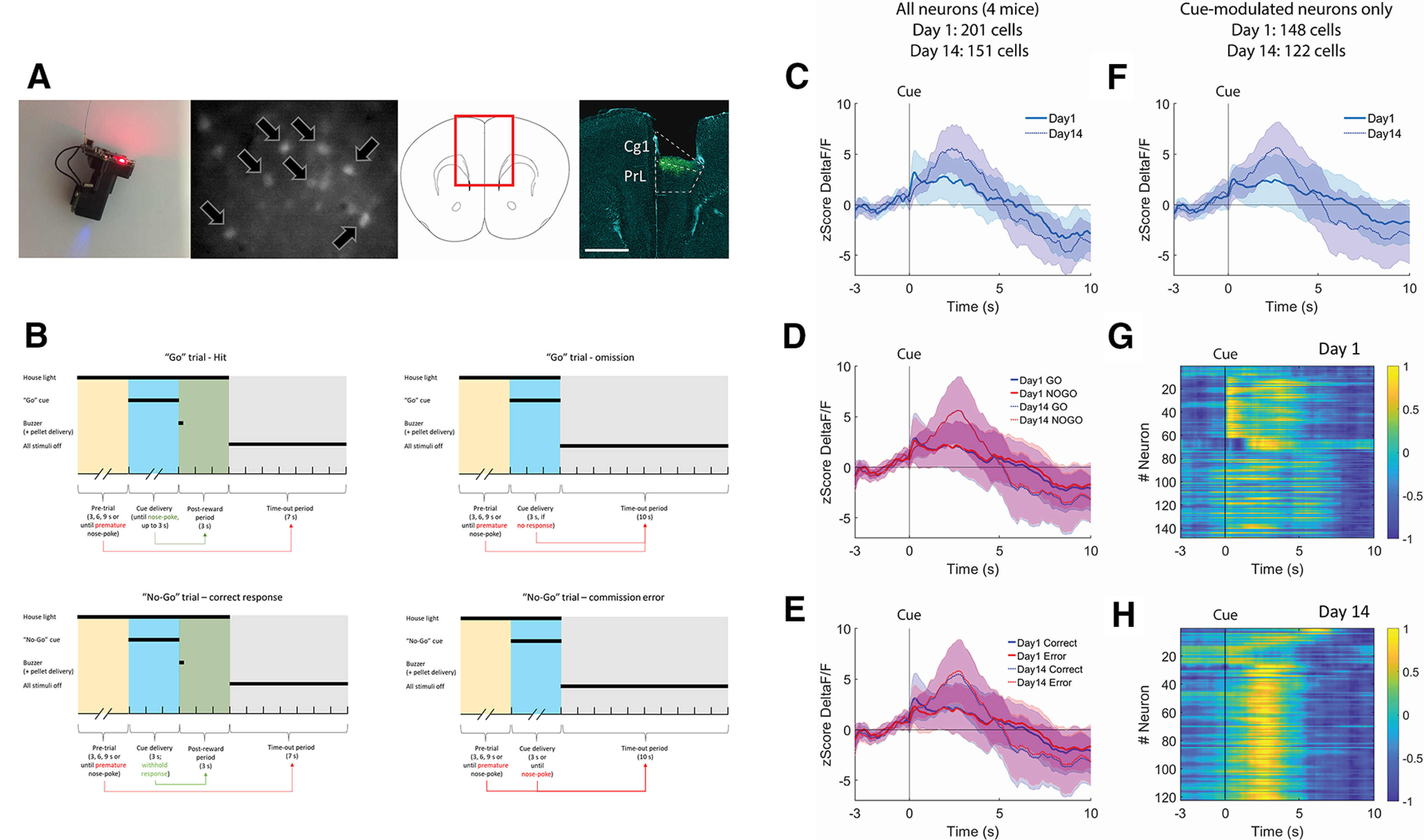
Calcium imaging. ***A***, The v3 UCLA Miniscope, a representative image of mPFC neurons expressing GCaMP6f (indicated by arrows), and a representative placement of the GRIN lens. Green immunofluorescence, GcaMP6f; cyan counterstain, DAPI. Cg1, cingulate cortex; PrL, prelimbic cortex. ***B***, A schematic representation of Go and No-Go trials depending on type of response. ***C***, Average neuronal activity across trials centered around the presentation of both Go and No-Go cues, on days 1 and 14 of the task. Data are shown as median (±MAD) z-scored change in fluorescence (ΔF/F), *n *=* *4. ***D***, The same data as in ***C***, showing Go and No-Go trials separately. ***E***, Same data showing the neuronal response on either correct or incorrect response in the trial (Go and No-Go trials are shown together). ***F***, The median z-scored change in fluorescence in response to Go and No-Go cues considering only significantly modulated neurons (Wilcoxon test, *p* < 0.0001). ***G***, ***H***, The same data as in ***F*** represented as change in fluorescence between 1 and −1, every row representing the fluorescence of a single neuron over time. Extended Data Figure 4-1 shows the Go/No-Go performance of the mice during the miniscope recordings.

10.1523/ENEURO.0430-21.2022.f2-4 Extended Data Figure 2-4 Arduino script for Go/No-Go training stage 1, left nose-poke hole active. Download Figure 2-4, TXT file.

10.1523/ENEURO.0430-21.2022.f2-5 Extended Data Figure 2-5 Arduino script for Go/No-Go training stage 1, right nose-poke hole active. Download Figure 2-5, TXT file.

10.1523/ENEURO.0430-21.2022.f2-6 Extended Data Figure 2-6 Arduino script for Go/No-Go training stage 2, left nose-poke hole active. Download Figure 2-6, TXT file.

10.1523/ENEURO.0430-21.2022.f2-7 Extended Data Figure 2-7 Arduino script for Go/No-Go training stage 2, right nose-poke hole active. Download Figure 2-7, TXT file.

10.1523/ENEURO.0430-21.2022.f2-8 Extended Data Figure 2-8 Arduino script for Go/No-Go training stage 3, left nose-poke hole active. Download Figure 2-8, TXT file.

10.1523/ENEURO.0430-21.2022.f2-9 Extended Data Figure 2-9 Arduino script for Go/No-Go training stage 3, right nose-poke hole active. Download Figure 2-9, TXT file.

10.1523/ENEURO.0430-21.2022.f2-10 Extended Data Figure 2-10 Arduino script for Go/No-Go training stage 4, left nose-poke hole active. Download Figure 2-10, TXT file.

10.1523/ENEURO.0430-21.2022.f2-11 Extended Data Figure 2-11 Arduino script for Go/No-Go training stage 4, right nose-poke hole active. Download Figure 2-11, TXT file.

#### Electronic components, Arduino circuit, and programming

The connectivity of the electronic circuit is presented in Figure 2*M*. Infrared LEDs and sensors detect nose-poke hole entries. There is a green LED above each nose-poke hole, and a house light for the presentation of visual stimuli. We installed two speakers for the presentation of auditory stimuli. We modified a Med Associates rat food pellet dispenser to administer food rewards (dustless chocolate-flavored pellets) by 3D-prinitng the internal component to accommodate mouse-sized food pellets. This choice of pellet dispenser was based on our available materials. A mouse pellet dispenser can be used directly without modifications and open-source alternatives are also available (see Discussion). The pellet dispenser was attached to the front wall of the chamber using screws. The Arduino microcontroller integrated and controlled all inputs and outputs of the circuit, including the start of the calcium imaging recording in the beginning of each behavioral session. We used the in-built port of the DAQ to send a triggering signal from the Arduino at the start of the behavioral testing session. This required a modification of the DAQ circuit board to include a SMA cable connection (Digikey, ID: WM9477-ND; see Table 2) to the Arduino (Extended Data Fig. 2-1).

**Table 2:** List of components necessary to build one operant chamber

Purpose	Item	Supplier/product number	Quantity	Price ($ US)
Arduino	IR break beam (for nose-poke holes)	Adafruit (ID: 2167)	2	2.33
circuit	Female DC plug	Adafruit (ID: 368)	1	1.58
	Diode 1N4001	Adafruit (ID: 755)	1	1.19
	Jumper wires	Adafruit (ID: 760)	1	6.28
	Speaker 2 (loud buzzer)	Ajax Scientific, rectangular buzzer with lead wire, 3V	1	3.16
	Resistor 700 ohm, 0.5W	Digikey (ID: CMF55700R00FKBF-ND)	1	0.11
	IRL 540 Transistor	Digikey (ID: IRL540PBF)	1	2.28
	Resistor 120 ohm, 0.5W	Mouser (ID: 588-OL1215E-R52)	2	0.17
	Resistor 10k ohm, 0.5W	Mouser (ID: 603-CFR-50JR-52-10K)	1	0.17
	White LED (very bright)	Mouser (ID: 630-ASMT-AY31-NUW01)	1	7.71
	Resistor 100 ohm, 0.5W	Mouser (ID: 660-CF1/2CT52A101J)	2	0.52
	Resistor 1k ohm, 0.5W	Mouser (ID: 660-CF1/2CT52R102G)	1	0.40
	28v power supply (for pellet dispenser) *	Mouser (ID: 709-GST25U28-P1J) *	1	*19.24
	Green LED	Mouser (ID: 941-C503BGCNCY0C0792)	2	0.28
	Speaker 1 0.5W (8 Ω)	SparkFun (ID: 09151)	1	1.78
	Arduino UNO R3	Sparkfun (ID: 11021)	1	18.13
	Breadboard	Sparkfun (ID: 12002)	1	3.91
			Total:	*72.55
For miniscope	Coaxial connector SMA	Digikey (ID: CONSMA013.062-ND)	3	5.80
and DAQ	Coaxial cable (for miniscope)	Digikey (ID: A9434W-10-ND)	1	30.22
	Coaxial cable (connections to DAQ)	Digikey (ID: A9432W-10-ND)	1	33.69
	Coaxial cable (DAQ modification)	Digikey (ID: WM9477-ND)	1	7.00
	PCB coaxial to SMA	https://oshpark.com/shared_projects/xtQGQ32E	5	0.59
			Total:	91.26
Other	Pipette tip box, lid removed	Cole-Parmer (ID: RK-25712-93)	1	30.26
components	Commutator *	Dragonfly R&D Inc. (ID: FL-2-C-Micro)*	1	*346.02
	Gorilla water-resistant tape	Home Depot CA (ID: 101593)	1	11.83
	Masking tape	Home Depot CA (ID: 2020–24)	1	1.16
	Pellet dispenser *	Med Associates*	1	*197.50
	USB camera	Newegg CA (ID: 9SIAVBFDST0991)	1	18.25
	UHU White tac	Staples CA (ID: 99683)	1	3.15
			Total:	*608.17
Chamber	Polycarbonate sheets (see Table 1)	Local plastics store	See Table 1	134.93
construction	SCIGRIP 16 plastics cement	SciGrip Adhesives (ID: 10319)	1	15.80
			Total:	150.73

Optional parts and prices that include them. Please see the discussion section for alternatives.

The Arduino scripts for each stage of the task are available as extended data (Extended Data Figures 2-2, 2-3, 2-4, 2-5, 2-6, 2-7, 2-8, 2-9, 2-10, 2-11). The scripts are named stage0 to stage4, the latter being the actual Go/No-Go task and all preceding stages being the training stages. All components necessary for the construction of one operant chamber are listed in Table 2.

## Results

### AcSD in adolescence leads to resilient and susceptible phenotypes in the SIT

Following exposure to AcSD in adolescence, susceptible mice had lower IRs (Fig. 3*A*) and spent less time in the IZ of the SIT (Fig. 3*B*) relative to controls. One-way ANOVAs revealed that resilient mice approached the social target similarly to controls (*n *=* *23; IR: *F*_(2,20)_ = 6.88, *p* = 0.0053, Holm–Sidak *post hoc* tests: control vs susceptible, *t*_(20)_ = 3.04, *p* = 0.0128, control vs resilient, *t*_(20)_ = 1.21, *p* = 0.2389; time spent in IZ: *F*_(2,20)_ = 7.68, *p* = 0.0034, Holm–Sidak *post hoc*: control vs susceptible, *t*_(20)_ = 2.85, *p* = 0.0197, control vs resilient, *t*_(20)_ = 1.81, *p* = 0.0852). Accordingly, there was a significant difference between resilient, susceptible and control mice in the time spent in corner zones of the SIT arena (*H*(2) = 6.51, *p* = 0.0386; Fig. 3*C*). As we have reported previously (Vassilev et al., 2021), there were more resilient than susceptible mice following AcSD in adolescence, 60% and 40%, respectively (Fig. 3*D*). This is a characteristic difference between the adolescent AcSD paradigm and the adult 10-d CSDS paradigm which leads to a majority of mice showing a susceptible phenotype (Krishnan et al., 2007; Golden et al., 2011).

### Exposure to AcSD in adolescence leads to impaired inhibitory control in adulthood as measured by the Go/No-Go task

In adulthood, approximately one month after the SIT (Fig. 3*E*), control and socially defeated mice were trained and tested on the Go/No-Go task (Fig. 3*F*). The Go/No-Go data were not divided according to the resilient and susceptible phenotypes observed in adolescence to increase the power of the statistical analysis and because we have previously shown that both groups perform similarly on the task (Vassilev et al., 2021). A 2 × 2 ANOVA revealed a significant main effect of time on the correct response rate (Fig. 3*G*) suggesting that, overall, mice improved their performance over time (*n *=* *17, *F*_(13,195)_ = 14.87, *p* < 0.0001). There was also a significant main effect of time on the proportion of commission errors (*F*_(13,195)_ = 13.85, *p* < 0.0001; Fig. 3*H*), indicating that, overall, mice became better at correctly withholding their response on No-Go trials. Finally, there was a significant TIME × STRESS group interaction effect on the proportion of commission errors (*F*_(13,195)_ = 1.92, *p* = 0.0303), suggesting that defeated mice showed inhibitory control impairment relative to controls, as they were less capable of withholding responding on No-Go trials. The main effect of time and the TIME × STRESS group interaction were non-significant for the proportion of hits (Fig. 3*I*), suggesting similar task engagement over time and between defeated mice and controls (TIME, *F*_(4.40,66.00)_ = 1.18, *p* = 0.3269; interaction, *F*_(13,195)_ = 1.67, *p* = 0.0689). These findings are in accordance with our previous findings showing cognitive impairments in adulthood following exposure to social defeat stress in adolescence in male mice (Vassilev et al., 2021).

### Miniscope calcium imaging reveals increased mPFC neuronal activity in response to Go and No-Go cues

To test whether our setup could be used for calcium imaging in freely moving mice, we implemented the UCLA Miniscope v3 to record calcium activity of mPFC neurons expressing GCaMP6f while mice (*n* =4) were performing the Go/No-Go task. The behavioral performance of the mice on the task is shown in Extended Data Figure 4-1. Histologic data revealed that the viral injection and the placement of the GRIN lens were targeted toward the anterior cingulate (Cg1) and prelimbic (PrL) areas of the mPFC (Fig. 4*A*). We calculated the median population activity of neuronal cells across trials on days 1 and 14 of the Go/No-Go task, within 3 s before and 10 s after the presentation of the Go and No-Go cues (Fig. 4*C–H*). This time window was chosen to include the minimum pretrial period and the minimum time following cue presentation until the next trial begins (Fig. 4*B*). Data are presented as median z-scored change in fluorescence signal (ΔF/F). We found an increase in overall activity from day 1 to day 14 following cue presentation (Fig. 4*C*). The same increase in activity from day 1 to day 14 was observed when we calculated population activity separately for Go and No-Go cues (Fig. 4*D*), suggesting that the increase in overall activity applied to both Go and No-Go trials. Finally, we observed the same increase in population activity when calculated separately depending on correct and incorrect responses across trials (Go and No-Go trials combined; Fig. 4*E*), suggesting that the increase in overall activity was not associated with task performance. The increase in overall activity was preserved if we selected only neurons that were significantly modulated by cue presentation (based on a Wilcoxon rank-sum tests, with a cutoff significance value at *p* < 0.0001; Fig. 4*F*). The heat-maps of individual cue-modulated neurons show that, on day 1 (Fig. 4*G*), cue presentation is followed by a diverse neuronal response with variable strength and timing between neurons, while on day 14 (Fig. 4*H*), cue presentation is followed by a more pronounced and synchronized neuronal activity.

10.1523/ENEURO.0430-21.2022.f4-1 Extended Data Figure 4-1 Go-No/Go performance of the 4 mice during calcium imaging. ***A***, Overall correct response rate. ***B***, Percent hits. ***C***, Percent commission errors. ***D***, Percent premature responses. Download Figure 4-1, TIF file.

## Discussion

Here, we present a low-cost, custom-built operant conditioning setup for the assessment of cognitive behavior in mice, paired with calcium imaging. Using our setup, we show that mice readily learn a Go/No-Go task, and that performance on this task is impaired in mice exposed to social defeat stress in adolescence, corroborating our previous findings (Vassilev et al., 2021). We demonstrate that our setup can be paired with a head-mounted miniscope for recording of calcium activity in freely moving mice by showing that neuronal activity in the mPFC increases from day 1 to day 14 of the task. We propose that our setup is a useful tool for the study of the neuronal activity correlates of both adaptive and pathologic behavior.

There are several advantages of our setup over other commercially available and open-source equivalents. It can be constructed with basic tools and materials within 2–3 d, and we include the Arduino code for the training and testing stages of the Go/No-Go task. Although our operant chamber uses only Arduino, which makes it easy to setup, it also offers the possibility to carry out complex tasks such as the Go/No-Go. This will allow users to recreate the behavioral testing setup quickly and easily, while also offering high flexibility and versatility. The chamber was constructed with an easily removable outer walls to facilitate cleaning, and we have found that the polycarbonate sheets are resistant to frequent cleaning with alcohol and other detergents. We are currently conducting parallel sessions with two operant chambers, as every miniscope requires its own desktop PC for optimal functionality. However, given enough available space, or other recording tools, our setup is easily scalable as it may be run even without direct connection to a PC. The Arduino can run on its own power supply and sessions can be restarted using the reset button. Finally, our operant setup allows for the reinforcement of nose-poking behavior, a widely used response in operant studies, while the mouse is carrying a head-mounted miniscope. Using our setup allows for adequate comparisons with other studies employing this type of reinforced response in mice, while adding the benefit of calcium imaging in freely moving animals. This is important considering documented differences between nose-poke and lever-pressing behavior in mice and rats (Schindler et al., 1993; Caine et al., 1999; Goeders et al., 2009). The fact that we replicated previous findings from our lab showing impaired performance on the Go/No-Go task in adulthood following social defeat stress in adolescence (Vassilev et al., 2021) demonstrates the reliability of our findings and the efficacy of our operant conditioning setup.

One aspect of our operant chamber that users might find difficult to reproduce is the adaptation of a Med Associates pellet dispenser for the delivery of food pellets. As an alternative to our approach, we suggest that the reader considers other custom-built pellet dispenser alternatives (Oh et al., 2017; O’Leary et al., 2018; Gurley, 2019). There are also several alternatives to our use of the Suite2p package for the analysis of calcium imaging data (Giovannucci et al., 2019; Pnevmatikakis, 2019; Cantu et al., 2020; Dong et al., 2021), and open-source alternatives to the UCLA Miniscope (Barbera et al., 2019; de Groot et al., 2020) and the DragonFly Inc. commutator (e.g., see miniscope.org; Barbera et al., 2020). One possible improvement on our setup would be the fabrication of a custom Arduino shield to improve the wiring stability of the electronic circuit. We did not include one with the current design to reduce the need for custom fabrication. Also, we used semi-transparent polycarbonate sheets for construction because of availability, and because we keep our boxes in sound-insulated cubicles but using opaque sheets for construction may reduce distractions for the mice during testing. Finally, we would like to point out that the design of our setup makes it possible to use it in conjunction with other techniques for imaging and manipulation of brain activity such as optogenetics and fiber photometry.

Creating new tools for the assessment of neural substrates of behavior is an indispensable part of neuroscience research. As accessible, open-source tools become more and more diverse, so do the possibilities to address specific, theory-driven questions. Our custom-built operant conditioning setup is a useful addition to the growing field of open-source science.

## Synthesis

Reviewing Editor: Tod E Kippin, University of California Berkeley Decisions are customarily a result of the Reviewing Editor and the peer reviewers coming together and discussing their recommendations until a consensus is reached. When revisions are invited, a fact-based synthesis statement explaining their decision and outlining what is needed to prepare a revision will be listed below. The following reviewer(s) agreed to reveal their identity: NONE.

The revised manuscript has now been re-evaluated by the original reviewers and both feel their prior major concerns have been addressed. As such, I believe the paper can be accepted for publication as long as the two minor concerns of Reviewer 1 regarding subheadings and statistics can be addressed (see below). Congratulations on the substantially improved manuscript.

Reviewer 1:

The authors mostly addressed my concerns.

I appreciate the revision of the parts list to allow adoption by other labs.

Minor suggestion: I noticed some of the subheadings are not very descriptive (e.g., “Go/Nogo”). The subheadings should be declarative statements in order to help the reader navigate the paper.

More importantly, I noticed some of the statistics are not properly reported. For example, for the “Social Interaction Test” and “Go/Nogo” sections of the Results, the F statistic and p values are reported, but Ns are missing and the statistical test is not described.

Other then those issues, I was satisfied with the revisions.

Reviewer 2:

The authors have adequately addressed my previous comments. I have no further concerns.

## Author Response

Where necessary, we have made corrections to the article manuscript, as per reviewers’ suggestions. These corrections are noted (page and line) after each of our replies below and are highlighted in yellow on the manuscript itself.

Reviewer 1:

The authors mostly addressed my concerns.

I appreciate the revision of the parts list to allow adoption by other labs.

Minor suggestion: I noticed some of the subheadings are not very descriptive (e.g., “Go/Nogo”). The subheadings should be declarative statements in order to help the reader navigate the paper.

We updated the headings of the methods and results sub-sections to describe better the contents of the article (p. 9 line 173; p. 10 line 191; p. 11 lines 211-212, 226-227; p. 12 lines 246-247).

More importantly, I noticed some of the statistics are not properly reported. For example, for the “Social Interaction Test” and “Go/Nogo” sections of the Results, the F statistic and p values are reported, but Ns are missing and the statistical test is not described.

We updated the “Social interaction test” and “Go/No-Go” subsections (after first changing the subsection headings) to better describe the statistical tests. We now clearly state the type of ANOVAs used and the type of post-hoc tests with the corresponding t-values and degrees of freedom. We also state the n’s for each analysis (p. 11, lines 214-219, p.12 lines 232-234).

Other then those issues, I was satisfied with the revisions.

Reviewer 2:

The authors have adequately addressed my previous comments. I have no further concerns.
