# Supplementary figures and images for: Custom-Built Operant Conditioning Setup for Calcium Imaging and Cognitive Testing in Freely Moving Mice
Source: eNeuro. 2022 Feb 9;9(1):ENEURO.0430-21.2022. doi: 10.1523/ENEURO.0430-21.2022 (PMC8856704; doi:10.1523/ENEURO.0430-21.2022)

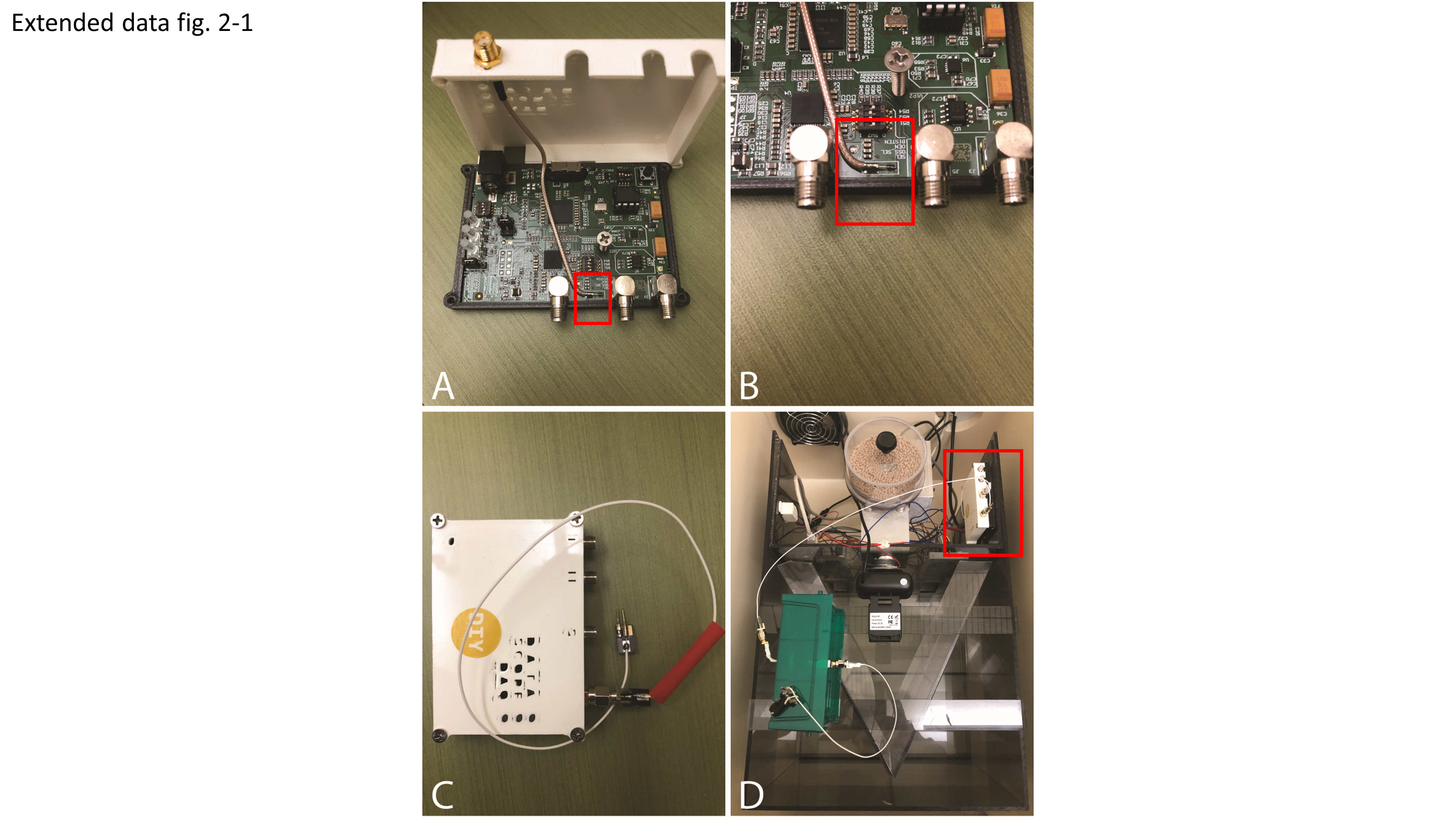

Supplement: Extended Data Figure 2-1 — Miniscope DAQ modification for external triggering of recording. A, B, A coaxial cable with an SMA connector is soldered to the input port (red square) right next to the miniscope input. C, A second coaxial cable is connected via SMA and a coaxial-to-SMA PCB. The free end is adapted to fit a breadboard by soldering two pins to the PCB. D, A top view of the operant chamber showing the miniscope DAQ (red square) connected to the miniscope via the coaxial cables (white) and the commutator. The placement of the USB camera is also visible. Download Figure 2-1, TIF file. [file enu-eN-OTM-0430-21-s03.tif]

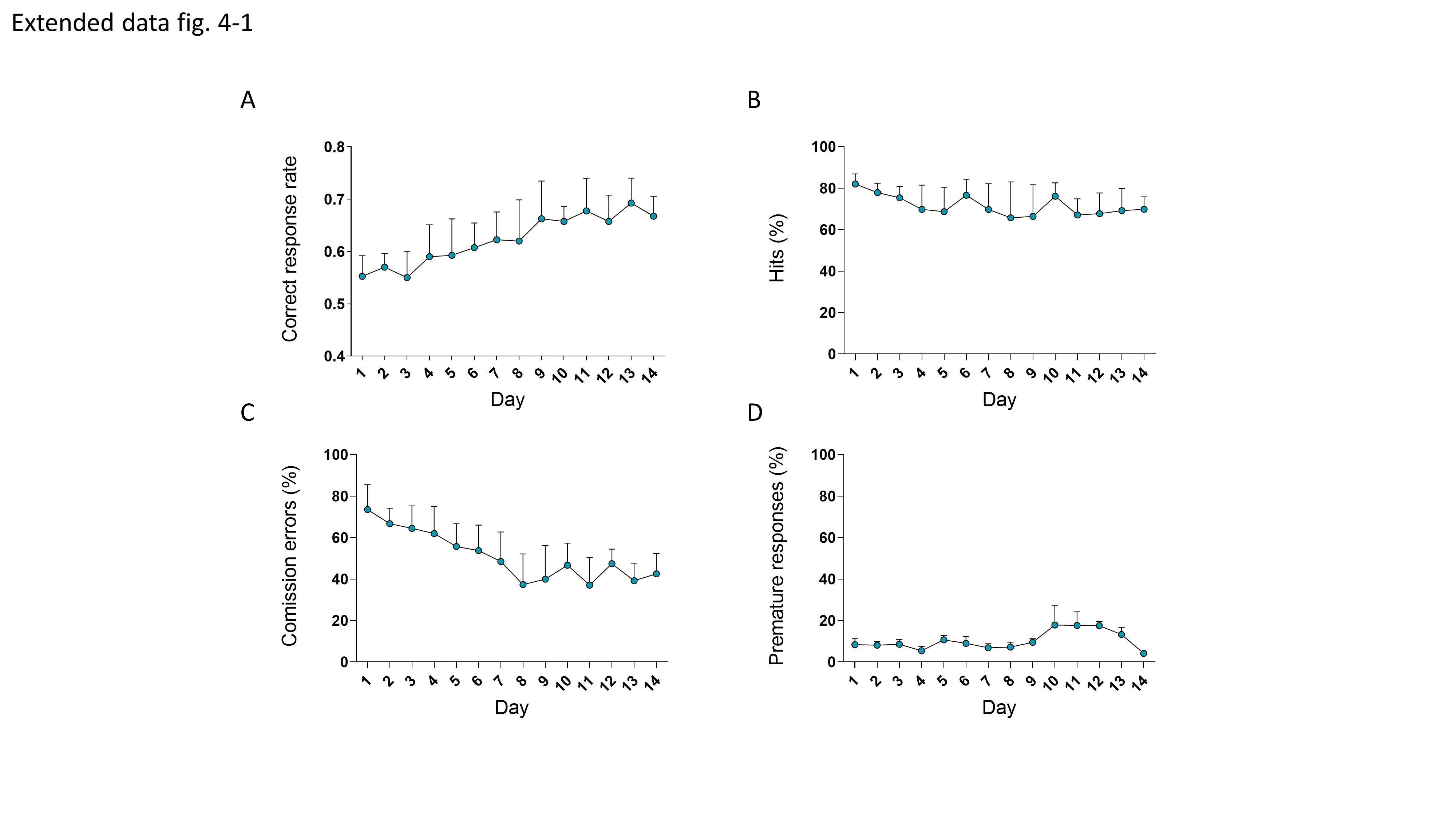

Supplement: Extended Data Figure 4-1 — Go-No/Go performance of the 4 mice during calcium imaging. A, Overall correct response rate. B, Percent hits. C, Percent commission errors. D, Percent premature responses. Download Figure 4-1, TIF file. [file enu-eN-OTM-0430-21-s04.tif]
